# A semi-automated robotic system for percutaneous interventions

**DOI:** 10.1007/s11548-023-02882-6

**Published:** 2023-04-14

**Authors:** Marius Siegfarth, Raffael Lutz, Nils-Christian Iseke, Javier Moviglia, Fabian Sadi, Jan Stallkamp

**Affiliations:** Mannheim Institute for Intelligent Systems in Medicine, Heidelberg University, Mannheim, Germany

**Keywords:** Medical robotics, Interventional radiology, CT robot, Percutaneous intervention

## Abstract

**Purpose:**

A robotic assistive device is developed for needle-based percutaneous interventions. The aim is a hybrid system using both manual and actuated robotic operation in order to obtain a device that has a large workspace but can still fit in the gantry opening of a CT scanner. This will enable physicians to perform precise and time-efficient CT-guided percutaneous interventions. The concept of the mechanics and software of the device is presented in this work.

**Methods:**

The approach is a semi-automated robotic assistive device, which combines manual and robotic positioning to reduce the number and size of necessary motors. The system consists of a manual rough positioning unit, a robotic fine positioning unit and an optical needle tracking unit. The resulting system has eight degrees of freedom, of which four are manual, which comprise encoders to monitor the position of each axis. The remaining four axes are actuated axes for fine positioning of the needle. Cameras are attached to the mechanical structure for 3D tracking of the needle pose. The software is based on open-source software, mainly ROS2 as robotic middleware, Moveit2 for trajectory calculation and 3D Slicer for needle path planning.

**Results:**

The communication between the components was successfully tested with a clinical CT scanner. In a first experiment, four needle insertions were planned and the deviation of the actual needle path from the planned path was measured. The mean deviation from the needle path to the target point was 21.9 mm, which is mainly caused both by translational deviation (15.4 mm) and angular deviation (6.8°) of the needle holder. The optical tracking system was able to detect the needle position with a mean deviation of 3.9 mm.

**Conclusion:**

The first validation of the system was successful which proves that the proposed concept for both the hardware and software is feasible. In a next step, an automatic position correction based on the optical tracking system will be integrated, which is expected to significantly improve the system accuracy.

## Introduction

Needle-based percutaneous interventions are used in clinical routine for a number of diagnostic and therapeutic procedures such as tissue biopsy or radiofrequency ablation [1, 2]. Although not yet established in clinical routine, approaches exist to support such procedures by robotic instrument guidance [3–9]. While some of those assistive devices are based on serial robotic arms as commonly used in industrial automation, an alternative approach is to develop devices that are specifically tailored to the geometric constraints of a computed tomography (CT) or magnetic resonance imaging (MRI) scanner, resulting in a cylindrical robot workspace. Examples of such devices are shown in [10, 11].

As the number of electric motors affects the size, weight and cost of a robotic system, the approach presented in this work is a semi-automated system that uses manual rough positioning and motorized fine positioning of the needle probe.

## Methods

The approach of our system is a semi-automated robotic assistive device, which combines manual and robotic positioning to place a needle and has a shape that fits in the gantry opening of a CT scanner. The aim is to obtain a system that has a large workspace spanning the whole patient’s body and high accuracy to place a needle based on target points selected on CT images, while the system must be designed to fit in the free space between the patient and CT scanner. To achieve this, the system is divided into three subsystems: a manual rough positioning unit, a robotic fine positioning unit and a needle tracking unit. The manual positioning unit is a mechanical device that allows the user to bring the needle holder close to the target position. By performing this step manually, large motors that would be necessary in a fully robotic system can be avoided and the system can be built more cost-efficiently. The robotic fine positioning unit is attached to the last joint of the manual positioning unit and is required for the needle to reach the target pose. Due to the low forces required to actuate the needle, this unit only requires small motors. The optical needle tracking unit is used to track the difference between the actual and target needle position without using X-ray imaging, to reduce the patient’s exposition to radiation. The measured error can then be compensated by the fine positioning unit. In addition to the hardware, a software is developed that enables both robot control and intervention planning.

**Fig. 1:**
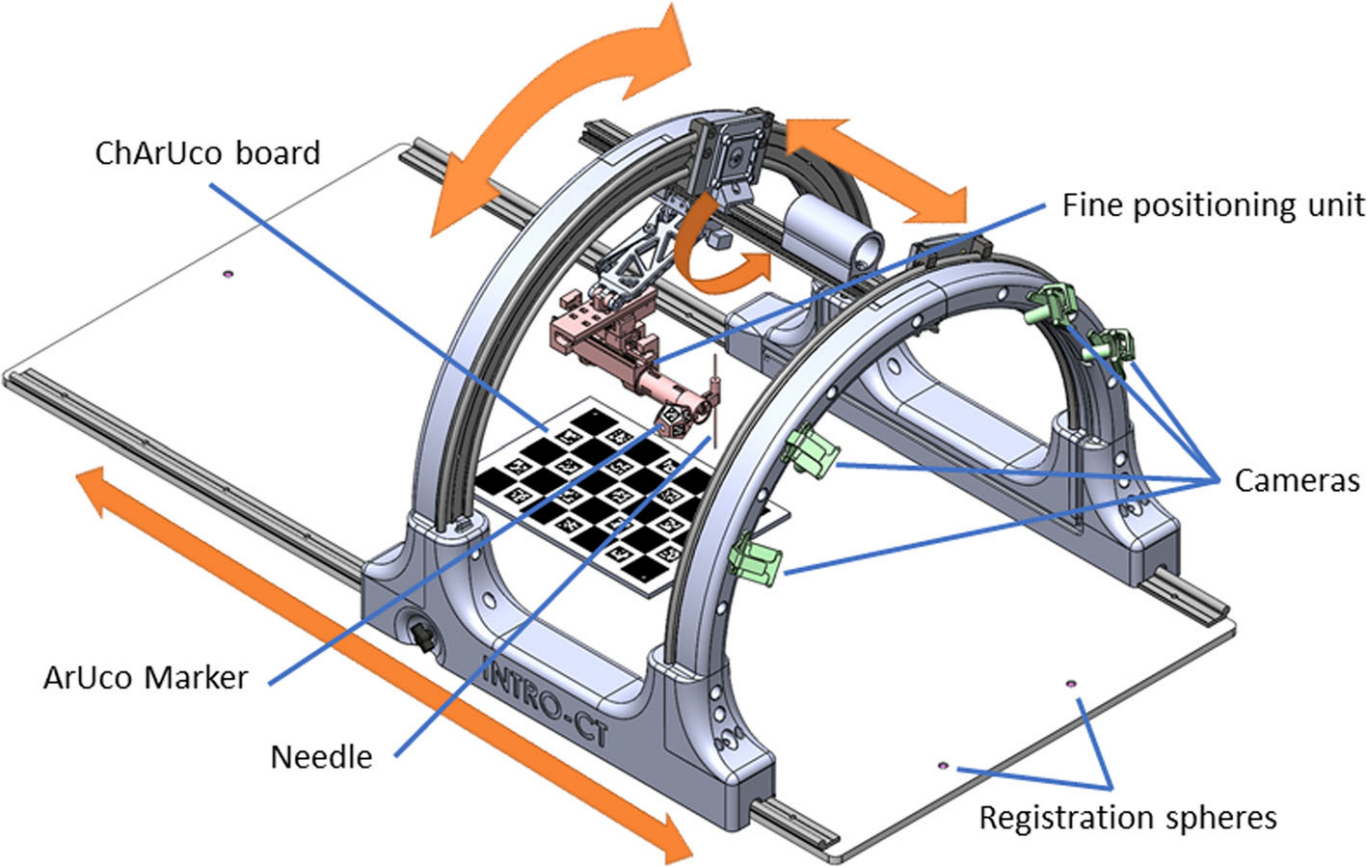
CAD drawing of the system. Arrows mark the manual movement along the rough positioning axes

A CAD drawing of the assistive device is shown in Fig. 1. The components are described in the following sections.

### Hardware

The device is set up as a manual rough positioning unit and a robotic fine positioning unit. It is controlled using a Raspberry Pi single board computer. The general design is an arch-like structure to fit in the gantry opening of a CT scanner. As the prototype cannot be integrated into the CT, it is built on a base plate which is placed on the CT table. Three marker spheres which are embedded in the base plate allow for automatic registration of the base position. The prototype is still shorter than the target system as it is only made to be used with a phantom instead of a whole body.

#### Rough positioning

The rough positioning unit consists of 4 axes: The first axis enables linear movement along the longitudinal axis, thus allowing the patient to lie on the table and the device being moved over the patient. The second axis is a curved linear guide to move the fine positioning unit on a circular trajectory around the patient. With the third axis, the fine positioning unit can be moved along the feet-head axis, in order to be able to avoid imaging artifacts by removing the fine positioning device from the imaging area during a scan. This allows the arch structure to remain in place, because materials that that are not prone to image artifacts, such as polymers and carbon fiber, were used for all components located within the imaging area. The fourth axis is a lever to place the fine positioning unit close to the patient.

The positions along the individual axes are measured by encoders. A multicolor LED light on each of the four axes provides visual feedback during manual rough positioning. Both encoders and LEDs are connected to the Raspberry Pi. When manually positioning an axis, the associated LED gradually changes color from red to green as the calculated target position is approached.

#### Fine positioning

The fine positioning unit consists of two linear and two rotational motors with encoders, allowing planar movement of the needle in two directions and angulation around two axes. Since the needle is advanced manually by the physician, all five necessary degrees of freedom are covered. The motors are located away from the needle to avoid imaging artifacts in the target structure, and to create free space for the manipulation of the needle by the physician. The mechanical design allows both in-plane and out-of-plane needle trajectories.

### Optical tracking system

Because the needle must be positioned and oriented in relation to the patient at the desired point as accurately as possible, a special needle tracking system may be required. Although it is possible to know the position of the needle with the encoders of each axis, there can be an error that cumulates throughout the kinematic chain. For this reason, an optical tracking system was developed. It consists of eight cameras placed along the arches of the structure connected to a Jetson Xavier NX board computer and a dodecahedron with ArUco markers located on the final axis of the fine positioning system.

The operating principle is similar to the one developed in [12]. Here a dodecahedron with markers on each of its faces is used in a passive stylus for real-time six degrees of freedom (6DoF) tracking, using only a monocular camera. The difference with our concept is that it is a multiview system, which provides a greater degree of redundancy in the event of a visual occlusion of one or more of the cameras.

The process of tracking the needle and registering it with respect to the tomographic image is described below.

Once the intrinsic parameters of each camera are found, the relative positions between each of them must be determined. For this purpose, a ChArUco board is used, which is positioned in a defined location on the base plate via two metal pins. The calibration is then performed by positioning the device along the first axis so that each camera sees at least one marker of the board. This process must be carried out only once, and at the time of calibration the encoder signal of the first axis must be recorded.

In this way, the transformation matrices between the dodecahedron and the cameras, the cameras and the ChArUco Board and the ChArUco Board and the ground plate can be calculated. The ground plate is registered in the CT by means of the spheres that are observed in Fig. 1. With this chain of transformations, the desired transformation of the tomographic image and the dodecahedron is obtained.

### Software

The software of the system is based on open-source software solutions: ROS2 is used [13] as robotic middleware, Moveit2 as robotic backend and 3D Slicer [14] as the frontend navigation software. The features of the mechatronic system such as motor control, encoder data acquisition, image acquisition, and kinematics calculations are implemented in ROS2 nodes which communicate over a local wired network. The 3D Slicer frontend software allows the user to display CT images and select target points to place a biopsy needle. 3D Slicer is connected to the ROS2 network using OpenIGTLink with the OpenIGTLinkIF plugin [12].

### Workflow

**Fig. 2:**
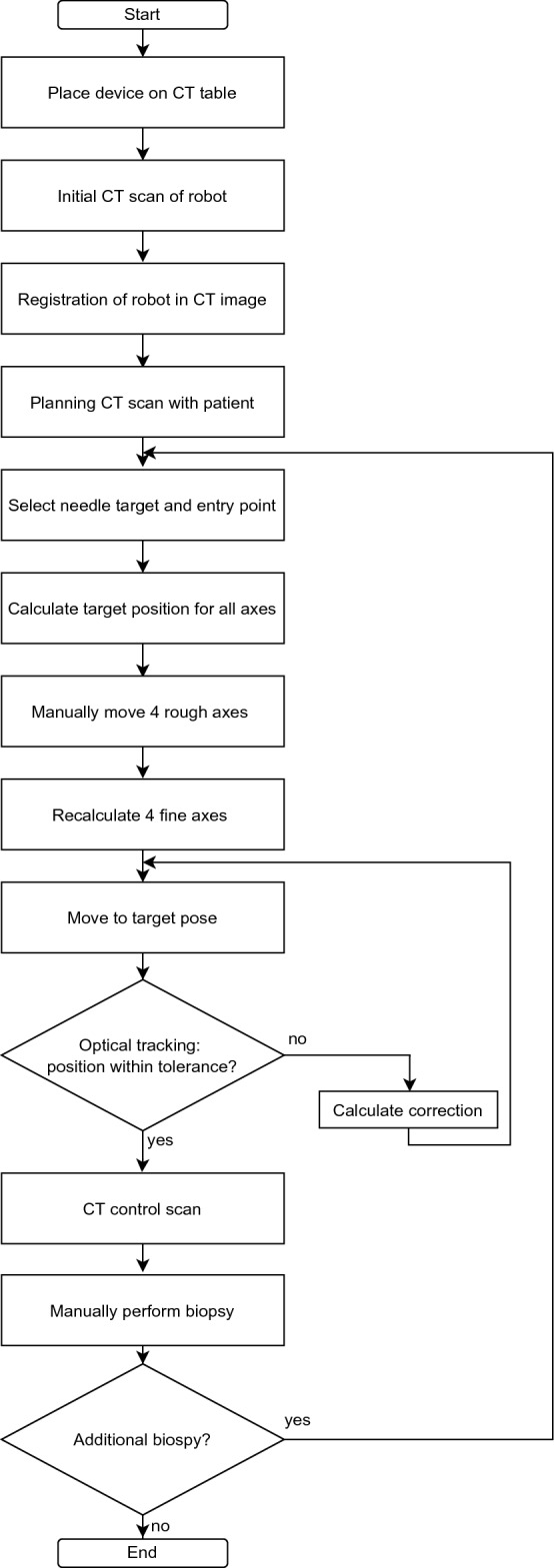
Workflow for a needle biopsy

For the process from registration of the robotic device to placing a needle, the following workflow is established (see Fig. 2):

The robot is placed on the CT table, and an initial registration scan is performed. The CT images are then transferred to the navigation software, and the robot is registered to the imaging coordinate system using the registration spheres. After registration, the robot’s coordinate system is identical to the scanner/image coordinate system.

For needle planning, the user manually selects the target and entry points in the navigation software. The coordinates of the points are transferred to the ROS2 network. By calculating inverse kinematics of the whole structure, the target position for each joint is determined. The user then moves the four manual axes to their respective target positions. Approximation to the target position is indicated by lights on each axis changing color from red to green once the position has reached a predefined tolerance. The position of the manual axes is locked by mechanical brakes. Inverse kinematics of the four actuated axes are then calculated again with the current values of the manual axes. The fine positioning unit then moves to the initial target joint positions.

Once the target position has been reached, the pose of the needle holder is tracked by the optical tracking system. If a deviation exists, a correction is calculated and the fine positioning can iteratively move until the target pose is reached.

## Results

**Fig. 3:**
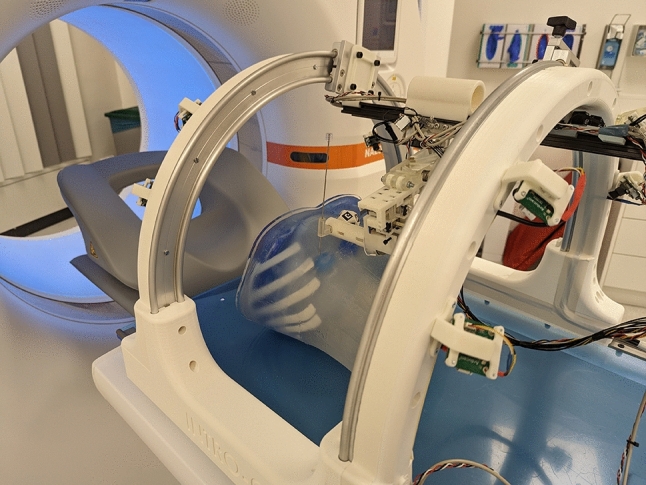
Robotic device on the CT scanner table

For an initial validation, the workflow was tested in a clinical environment, as shown in Fig. 3. The system was placed on the table of a CT scanner together with an abdominal phantom including liver lesions, further described in [15].

The system was registered to the patient table, and an initial scan of the phantom was recorded. For each of four experiments, the needle path to a lesion in the liver was defined by selecting one target point and one entry point in the navigation software. The coordinates of the needle path were then sent to the robot system. When the system had calculated the target joint positions, we manually moved the four rough positioning axes to the target positions, guided by the respective LEDs. Afterward, the target position of the fine positioning axes was recalculated and the fine positioning unit moved the needle holder to the target needle path. The final position of the needle holder was recorded in a CT scan, and the scan was loaded into the navigation software. We manually selected the actual needle path from the CT images and compared it to the planned path. The result was a mean angular deviation of 6.8° (6.6, 6.0, 7.4, 7.4) and the spatial distance of the central needle holder point from the planned needle path was 15.4 mm (9.4, 15.4, 17.4, 19.2). The distance between the target point and the actual needle path is 21.9 mm (15.0, 20.9, 26.7, 25.0), which is a result of both the translational and rotational error. The main source of the translational error was identified as the inaccuracy of the encoder in the first manual axis. To simulate the accuracy that would be possible without this error, we replaced the encoder position with the true position of the first axis. This resulted in reduction of the mean spatial distance between needle holder and needle path to 3.4 mm.

As automatic position correction using the optical tracking system did not yet run stably, we used the optical system only to measure the needle holder position and calculated the deviation from the true needle holder position, obtained from the CT images. The result was a mean deviation of 3.9 mm (3.8, 3.6, 3.8, 4.2). In this experiment, the needle holder orientation could not be determined, as the number of ArUco markers simultaneously captured by the cameras was too low for a stable angular measurement.

## Discussion

We proposed a semi-automated device to assist the placing of needles in a CT scanner for interventional radiology. The resulting system is a hybrid device comprising manual degrees of freedom that are operated by the user and robotic degrees of freedom for automated fine positioning. Both the mechanical setup and the software architecture were validated in a clinical CT scanner, where the communication between software components proved to work as required. This was especially important for the software interfaces, since in our concept the CT imaging, robot control, navigation software and needle tracking run on different computers and are connected through a network.

A first experimental validation showed that the deviation of the needle path from the planned path is still higher than it would be required in order to accurately place a needle in a small lesion. However, the optical tracking system was able to detect the actual needle position with higher accuracy than the internal encoders, so we expect to obtain an accuracy improvement by implementing automatic position correction based on the optical measurement. To obtain stable measurements for both the translation and orientation using ArUco markers, we will have to increase the number of visible ArUco markers. In addition, the position encoders in all axes and their respective calibration will be improved in a next development step to further improve the geometric accuracy.
